# Gut Microbiome Changes Associated with Epithelial Barrier Damage and Systemic Inflammation during Antiretroviral Therapy of Chronic SIV Infection

**DOI:** 10.3390/v13081567

**Published:** 2021-08-08

**Authors:** Ceylan Tanes, Edith M. Walker, Nadia Slisarenko, Giovanni L. Gerrets, Brooke F. Grasperge, Xuebin Qin, S. Michal Jazwinski, Frederic D. Bushman, Kyle Bittinger, Namita Rout

**Affiliations:** 1 Division of Gastroenterology, Hepatology and Nutrition, Children’s Hospital of Philadelphia, Philadelphia, PA 19104, USA; tanesc@email.chop.edu (C.T.); bittingerk@email.chop.edu (K.B.); 2 Division of Microbiology, Tulane National Primate Research Center, Tulane University, Covington, LA 70433, USA; ewalker5@tulane.edu (E.M.W.); nslisare@tulane.edu (N.S.); Giovanni.Gerrets@crl.com (G.L.G.); 3 Division of Veterinary Medicine, Tulane National Primate Research Center, Tulane University, Covington, LA 70433, USA; bgrasper@tulane.edu; 4 Division of Comparative Pathology, Tulane National Primate Research Center, Tulane University, Covington, LA 70433, USA; xqin2@tulane.edu; 5 Tulane Center for Aging, Tulane University School of Medicine, New Orleans, LA 70112, USA; sjazwins@tulane.edu; 6 Department of Microbiology, Perelman School of Medicine, University of Pennsylvania, Philadelphia, PA 19104, USA; bushman@pennmedicine.upenn.edu

**Keywords:** SIV, ART, macaques, microbiome, IFABP, sCD14, LBP

## Abstract

Gut dysbiosis is a common feature associated with the chronic inflammation of HIV infection. Toward understanding the interplay of chronic treated HIV infection, dysbiosis, and systemic inflammation, we investigated longitudinal fecal microbiome changes and plasma inflammatory markers in the nonhuman primate model. Following simian immunodeficiency virus (SIV) infection in rhesus macaques, significant changes were observed in several members of the phylum Firmicutes along with an increase in Bacteroidetes. Viral suppression with antiretroviral therapy (ART) resulted in an early but partial recovery of compositional changes and butyrate producing genes in the gut microbiome. Over the course of chronic SIV infection and long-term ART, however, the specific loss of *Faecalibacterium prausnitzii* and *Treponema succinifaciens* significantly correlated with an increase in plasma inflammatory cytokines including IL-6, G-CSF, I-TAC, and MIG. Further, the loss of *T. succinifaciens* correlated with an increase in circulating biomarkers of gut epithelial barrier damage (IFABP) and microbial translocation (LBP and sCD14). As *F. prausnitzii* and *T. succinifaciens* are major short-chain fatty acid producing bacteria, their sustained loss during chronic SV-ART may contribute to gut inflammation and metabolic alterations despite effective long-term control of viremia. A better understanding of the correlations between the anti-inflammatory bacterial community and healthy gut barrier functions in the setting of long-term ART may have a major impact on the clinical management of inflammatory comorbidities in HIV-infected individuals.

## 1. Introduction

Persistent inflammation during HIV infection is strongly associated with gut dysbiosis [1,2,3], and contributes to a high risk of morbidity and mortality in persons living with HIV [4]. The significant shifts in gut microbiota of HIV-infected persons remain unresolved despite initiation of antiretroviral therapy (ART) and are interrelated with intestinal barrier damage and altered mucosal immune responses [5,6]. As first demonstrated in the SIV-infected macaque model [7] and later confirmed in HIV-infected humans [8,9], it is well established that HIV rapidly infects and depletes gastrointestinal CD4^+^ T cells within days of infection regardless of the route of exposure. The sustained loss of CD4^+^ T cells, particularly the epithelial barrier protective mucosal CD4^+^ T-helper 17 (Th17) cells is linked to microbial translocation (MT) and persistent inflammation despite continued ART [10,11,12,13]. Together, unresolved gut dysbiosis, loss of gut barrier integrity, MT, and chronic immune activation contribute to the persistent inflammation during chronic HIV infection. Thus, a better understanding of the host–microbe interaction at the gut mucosal interface is essential to develop therapeutic approaches toward alleviating chronic inflammation in ART-treated HIV-infected persons.

Multiple HIV and SIV studies have assessed the associations between changes in gut microbiome and pathogenesis [14,15,16,17]. Yet, the impacts of acute viremia and subsequent long-term ART-treated chronic HIV infection on the microbial composition remain poorly understood. Cross-sectional studies have revealed shifts in the relative abundance of the genera Bacteroides and Prevotella in the gut microbiome associated with immune activation and disease progression [15,17,18,19]. Conversely, other studies showed no difference in Bacteroides and Prevotella [20,21], likely due to inherent differences in study populations such as lifestyle, diet, and sexual practice. Indeed, the latter has been shown to have a greater influence on the gut microbiome than HIV status or ART regimen [22,23,24]. Thus, longitudinal studies are essential to determine cause-and-effect relationships between the host microbiome composition and systemic inflammation. Due to practical challenges and numerous confounding factors of human studies, longitudinal data collected over a period of untreated or ART-treated infection are mainly derived from the SIV-macaque model [25,26,27,28,29,30].

Although the gut microbial composition of macaques is slightly different from humans [31], the dynamic changes in gut microbiome and its interrelationship with gut immunity and inflammation following SIV infection and through the course of long-term ART can provide important information on how specific bacterial species may impact the persistent inflammation of chronic treated HIV infection. However, longitudinal studies on the impact of chronic HIV/SIV infection with ART on gut microbiome rarely extend beyond one year. Here, we investigated the fecal microbiome composition within individual rhesus macaques before and during SIV infection and with up to 21 months of effective treatment with ART. We further examined circulating markers of systemic inflammation, intestinal epithelial barrier damage (IEBD), and microbial translocation (MT) during this period to determine the impact of ART on the restoration of gut microbial shifts caused by SIV infection and the dynamic changes during inflammation of chronic SIV-ART.

## 2. Materials and Methods

### 2.1. Ethics Statement

Animals in this study were housed at the Tulane National Primate Research Center (TNPRC). The institution has received continued full accreditation by the Association for Assessment and Accreditation of Laboratory Animal Care (AAALAC) International. The study was approved by the Tulane University Institutional Animal Care and Use Committee (IACUC; P0359-2017) and was conducted under the standards of the US National Institutes of Health Guide for the Care and Use of Laboratory Animals. The NIH Office of Laboratory Animal Welfare assurance number for the TNPRC is A4499-01. Following SIV infection, animals were housed in Animal Biosafety Level 2 indoor housing. All animal procedures including virus administration, sample collection, and euthanasia were carried out under the direction of TNPRC veterinarians.

### 2.2. Animals, Viral Inoculation, and ART

Six healthy female Indian ancestry rhesus macaques ranging in age from 5 to 10 years old and seronegative for SIV, HIV-2, STLV-1 (Simian T Leukemia Virus type-1), SRV-1 (type D retrovirus), and herpes-B viruses were used in this study. MHC-1 genotyping for the exclusion of the common Mamu alleles Mamu-A*01/-A*02 and Mamu-B*08/-B*17 was performed by sequence-specific priming PCR. The macaques were socially housed in pairs after enrolment in the project. Animals were infected with 2500 TCID50 SIV_mac_251 via the intrarectal (IR) route using the pathogenic SIV challenge stocks obtained from the Preclinical Research and Development Branch of Vaccine and Prevention Research Program, Division of AIDS, NIAID. cART consisted of daily subcutaneous injections of 5.1 mg/kg Tenofovir Disoproxil Fumarate (TDF), 30 mg/kg Emtricitabine (FTC) and 2.5 mg/kg Dolutegravir (DTG) in a solution containing 15% (*v*/*v*) kleptose at pH 4.2, as previously described [32].

### 2.3. Plasma and Fecal Sample Collection

Blood samples in K_2_-EDTA anticoagulant coated tubes (Sarstedt Inc. Newton, NC, USA) were taken for a complete blood count and routine chemical analysis and centrifuged for plasma separation. Approximately 250 mg of fresh fecal sample from each animal was collected using a sterile fecal loop into a sterile 2 mL cryovial and stored at −80 °C until use for genomic DNA extraction. Longitudinal samples were collected from pre-SIV, one month following SIV infection, and during ART at various time-points between 2 and 21 months from 6 SIV-infected treated rhesus macaques. Plasma viral load quantification was performed using a Roche High Pure Viral RNA kit (Catalog #11858882001) as previously described [33].

### 2.4. Plasma Markers of Inflammation, Microbial Translocation, and Intestinal Damage

Plasma samples were thawed and cleared using Ultrafree centrifugal filters (Millipore, Billerica, MA, USA). Cytokines, chemokines, and growth factors were quantified in plasma using the Non-Human Primate Cytokine/Chemokine/Growth Factor 37-Plex ProcartaPlex Panel (Invitrogen, Life Technologies, Waltham, MA, USA), following the manufacturer’s instructions. Data were acquired with a Bio-Plex 200 analyzer and analyzed using Bio-Plex Manager software v6.1 (Bio-Rad, Hercules, CA, USA). Commercially available Monkey IFABP/FABP2 and LBP ELISA kits (MyBioSource, San Diego, CA, USA) were used to quantify the intestinal fatty acid binding protein (IFABP) and LPS-binding protein (LBP) in plasma samples. The commercially available ELISA kit for Human sCD14 (R&D Systems, Minneapolis, MN, USA) was used according to the manufacturer’s protocols with 1:200 dilution of plasma samples. All assays were performed in duplicate, and data were analyzed using Gen 5 software (BioTek, Winooski, VT, USA).

### 2.5. DNA Purification, Library Preparation, and Sequencing

DNA was extracted from approximately 250 mg of stool using the Qiagen DNeasy PowerSoil kit (Qiagen, Germantown, MD, USA). Extracted DNA was quantified with the Quant-iT PicoGreen_dsDNA kit (Thermo Fisher, Waltham, MA, USA). Libraries for shotgun metagenomic sequencing were generated using the NexteraXT kit and sequenced on the Illumina HiSeq 2500 instrument. Extraction blanks and DNA-free water were included to empirically assess environmental and reagent contamination.

### 2.6. Sequencing Data Analysis

Shotgun metagenomic sequence data were processed using the Sunbeam metagenomics pipeline [34]. Trimmomatic was used to trim Illumina adapter sequences and low-quality basecalls [35]. Host DNA was identified by alignment using Burrows-Wheeler Alignment tool (BWA) [36], and low-complexity sequence reads were removed using the Komplexity tool [34]. Reads mapping to the PhiX genome were also removed. The abundance of bacteria was estimated using MetaPhlAn software v3.0 [37]. Sample similarity was assessed using the Bray–Curtis distance and alpha diversity was assessed using the Shannon diversity metric. Reads were mapped to the KEGG protein database [38] to estimate the abundance of bacterial gene orthologs using RAPSearch2 [39].

### 2.7. Statistical Analysis

Comparisons for plasma analytes were performed using GraphPad Prism software version 8.4.3 (GraphPad, San Diego, CA, USA). Data were analyzed by one-way ANOVA with multiple comparisons with a test for linear trends, or two-way ANOVA with repeated measures. Tukey’s and Dunnett’s post hoc tests were used for multiple comparisons. *p* < 0.05 was considered significant. For bacterial abundance, the differences across time in qPCR levels and Shannon diversity were assessed using linear mixed effects models with the study time-points as fixed effects and the animal IDs as random effects. The cohousing information of the animals was added as a covariate to the model. Community level differences between sample groups were assessed using the permutational multivariate analysis of variance (PERMANOVA) test. Bacterial species and gene abundance levels were tested using linear mixed effects models on logit transformed relative abundances. When multiple tests were performed, the p-values were corrected for a false discovery rate using the Benjamini–Hochberg method. The correlations between the plasma cytokines and bacterial relative abundances were assessed using linear mixed effects models with the animal IDs as random effects. The cohousing information was added as a covariate to all the tests.

## 3. Results

### 3.1. Coordinated Increase in Systemic Inflammation and Gut Epithelial Barrier Dysfunction during Long-Term SIV-ART in Rhesus Macaques

**Figure 1:**
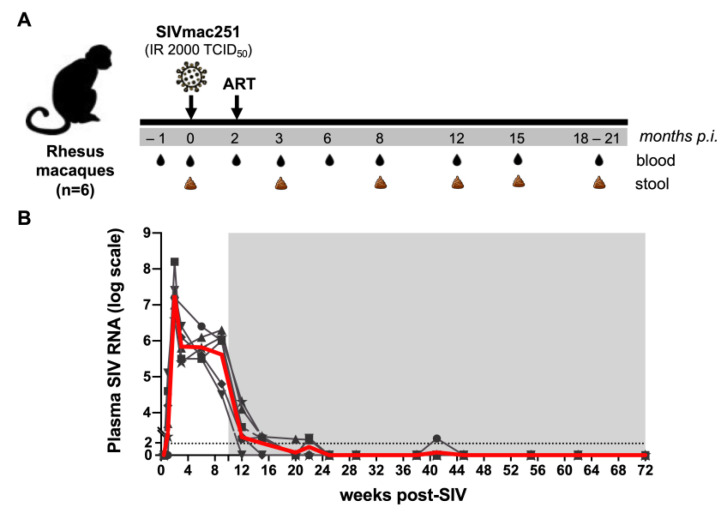
Study schedule and plasma viral loads. (**A**) Six animals were treated with a combination of ART drugs TDF, FTC, and DTG. Baseline samples were collected on d0 SIV, and ART was started 10 weeks following SIV infection. (**B**) Longitudinal SIV RNA quantification in plasma for 72 weeks of SIV-ART. The grey area represents ART. The red line is the average viral load for all animals.

To understand the coordinated roles of intestinal barrier function and gut microbiota in systemic inflammation during long-term antiretroviral therapy, we examined circulating IEBD and MT biomarkers, inflammatory cytokines, and gut microbial communities in a cohort of six SIV-infected macaques through the course of SIV-ART. The study design is shown in Figure 1A. As expected, following SIV infection, plasma viremia peaked at 2 weeks of infection to an average 7.2 log10 copies/mL and by 4 weeks had reduced by 2 logs and settled at ~5.8 log10 copies/mL (Figure 1B).

Early SIV infection resulted in a significant increase in plasma levels of multiple inflammatory cytokines and chemokines including IL-1β, IL-18, IL-1Rα, CXCL9/monokine induced by gamma (MIG), CXCL11/Interferon-inducible T cell alpha chemoattractant (I-TAC), macrophage inflammatory protein (MIP)-1β, Granulocyte colony stimulating factor (G-CSF), and CXCL13 (Supplementary Figure S1A). As we have recently reported [40], this was accompanied with elevated levels of the leaky gut and microbial translocation (MT) markers IFABP and LBP (Supplementary Figure S1B), in agreement with several other studies revealing intestinal barrier disruption during acute HIV/SIV infections [41,42,43]. This also coincided with a significant decline in frequencies of peripheral CD4 T lymphocytes (data not shown). Following set-point viremia, the animals were treated daily with the three-drug ART regimen that resulted in the stable suppression of viremia (Figure 1B). Effective viral suppression resolved the plasma inflammatory cytokines and leaky gut biomarkers by 3 months (3 mo) of treatment (Supplementary Figure S1). However, despite continued ART and initial resolution of IEBD and MT, an increase in IFABP and LBP was observed at the 8 month ART time-point. Further, there was a significant increase in plasma inflammatory cytokines including GM-CSF, MCP-1, IP-10, IL-6, IFN-γ, IL-12, and TNF-α (Supplementary Figure S1). This suggested a synchronized development of leaky gut and systemic inflammation during chronic infection regardless of long-term suppression of viremia in the SIV-infected macaques under ART.

### 3.2. Impact of SIV Infection on the Relative Abundance of Gut Bacteria

**Figure 2:**
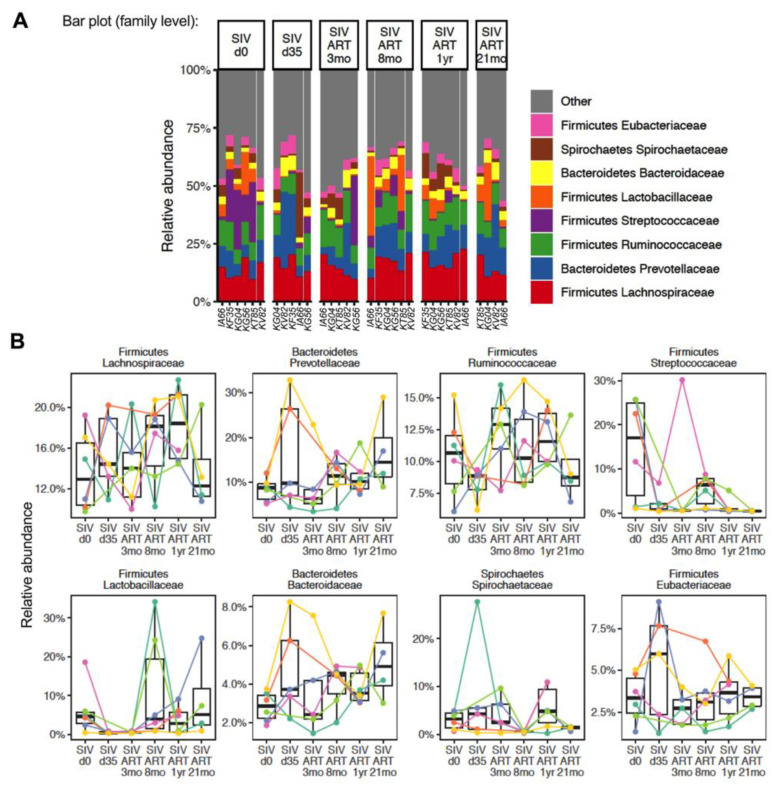
The composition of gut bacterial communities at the family level. (**A**) Relative abundance of fecal bacterial taxa at the family level in each animal at baseline (d0), d35 post-SIV infection, and at 3 mo, 8 mo, 12 mo, and 21 mo ART time-points. (**B**) Longitudinal changes in relative abundance in individual animals at the indicated time-points. IA66 and KT85; KF35 and KG56; KG04 and KV82 were pair-housed to enable social housing.

Consistent with previous reports [29,44], the baseline fecal microbiome prior to the SIV challenge was composed predominantly of Firmicutes and Bacteroidetes. Spirochaetes, represented by *Treponema*, were the only other phylum to appear with over 1% relative abundance. The Firmicutes were a mixture of facultative anaerobes (*Streptococcus*, *Lactobacillus*) and obligate anaerobes (*Faecalibacterium*, *Ruminococcus*, *Eubacterium*) (Figure 2).

**Figure 3:**
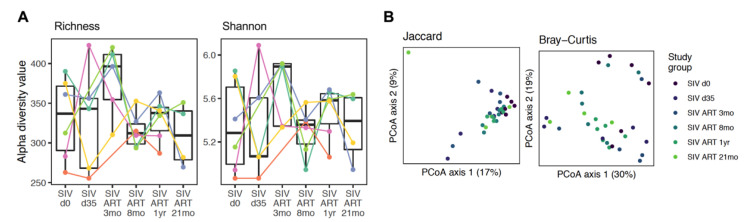
Diversity in fecal samples. (**A**) Alpha diversity, measured by observed species in the Richness and Shannon index, is plotted for animals at baseline (SIV.d0), at d35 post-SIV infection, and at 3 mo, 8 mo, 12 mo, and 21 mo ART. Each animal is represented by a different color. The line inside the box represents the median, while the whiskers represent the lowest and highest values. Linear mixed effects models showed no difference for species with Richness or Shannon diversity between the time-points. (**B**) Principal coordinates analysis (PCoA) plots of Jaccard and Bray–Curtis distances to assess beta diversity at different time-points of SIV-ART. No statistically significant differences were observed between the different ART time-points using the PERMANOVA test.

The baseline composition and kinetics of changes through the course of SIV-ART varied greatly between individuals (Figure 2B). To determine the contribution of early viral replication prior to suppression with ART, we first compared the bacterial composition in fecal samples between the baseline and at d35 post-SIV infection. At d35 post-SIV, we did not observe a difference from the baseline in alpha diversity (Figure 3A). The community structure was different from the baseline after 8 months based on the PERMANOVA test on Bray–Curtis distances (*p* = 0.03) (Figure 3B).

**Figure 4:**
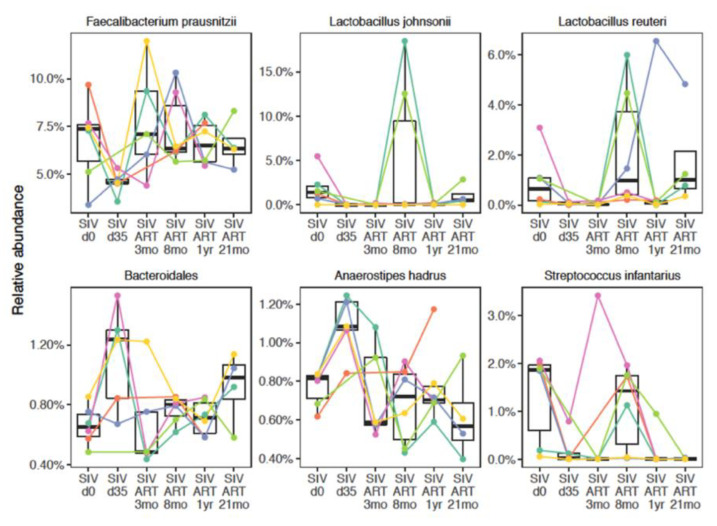
Relative abundance at species level through the course of SIV-ART. Bacterial species showing either a reduction in abundance at d35 post-SIV infection compared to baseline (SIV.d0), and recovery at 3–8 months of ART, or an increase in abundance at d35 post-SIV infection compared to baseline (SIV.d0) and a return to baseline at 3–8 months of ART.

In an exploratory analysis of species level relative abundance, we observed a decrease in *Lactobacillus johnsonii* (*p* = 0.011), *Faecalibacterium prausnitzii* (*p* = 0.028), and *Streptococcus infantarius* (*p* = 0.042) following SIV infection (Figure 4), without correcting for multiple comparisons.

Conversely, the relative abundance of *Anaerostipes hadrus* increased (*p* = 0.00032) at this time-point (Figure 4). However, after correcting for multiple comparisons using the Benjamini–Hochberg method to control for a false discovery rate of 5%, a statistically significant difference was observed only for *A. hadrus* (fdr = 0.012).

**Figure 5:**
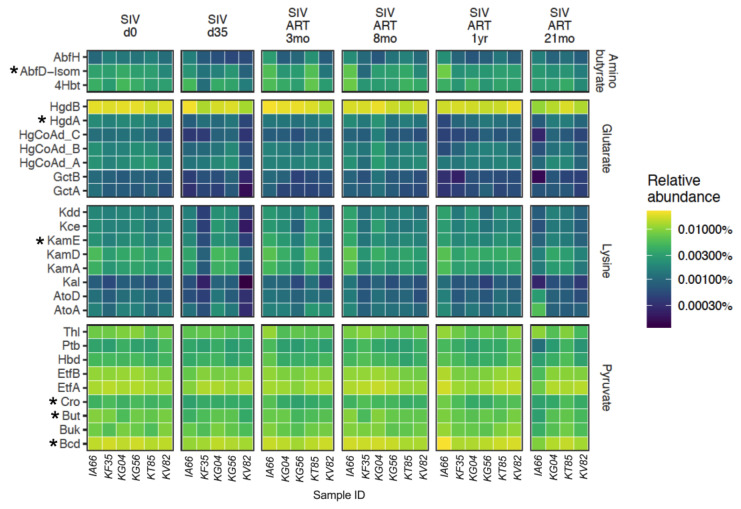
Changes in butyrate producing genes. The change in relative abundance of genes in four pathways responsible for butyrate production as curated by Vital et al. [42]. Rows represent individual genes, and the columns represent individual animals at each study time-point. Asterisks represent if the gene shows a statistically significant linear decrease through SIV infection and ART based on linear mixed effects models. The final time-point of 21 mo ART has only four animals since two of the study animals were euthanized at the 18 mo ART time-point.

We next examined microbial gene abundances to gain a better understanding of the potential for metabolite production. Of particular interest was butyrate, which is primarily synthesized following fermentation of dietary fiber and plays an important role in maintaining gut homeostasis [45]. We examined gene abundances in four butyrate-production pathways: one based on carbohydrate metabolism (pyruvate), and three involving amino acid metabolism (lysine, aminobutyrate, and glutarate). Several genes in the amino acid pathways were significantly decreased following SIV infection (Figure 5), including AbfD-Isom, HgdA, KamE, Cro, Bcd, and But (fdr = 0.024), indicating that SIV-induced alterations in gut bacterial abundances impacted the production of metabolites essential for intestinal epithelial cell function.

### 3.3. Longitudinal Progression of Gut Microbiome during ART

To understand the impact of viral suppression with ART on the stabilization of SIV-induced perturbations, we then examined the longitudinal progression of fecal microbial composition in the SIV-challenged macaques. Initiation of ART was associated with microbiome differences in the cohort. Again, we carried out an exploratory time-series analysis to identify recovery signatures during ART. This analysis revealed a rebound in the abundance of *Streptococcus infantarius* (*p* = 0.047), and *Lactobacillus reuteri* (*p* = 0.0087) by 3–8 months of continuous ART (Figure 4). Conversely, the acute increase in *Anaerostipes hadrus* and *Bacteroidetes bacteroidales* at 1 month post-SIV infection returned to baseline levels by 3 months of ART (*p* = 0.00032 and 0.046 respectively), showing a trend toward early signs of recovery with control of viremia (Figure 4). However, the evidence for recovery in these taxa was not statistically significant after controlling for a false discovery rate of 5% across all comparisons, underscoring the exploratory nature of the species level results.

Since we observed an increase in immune activation and microbial translocation in our cohort around 8 months of ART, we next examined the changes in the butyrate producing potential of the microbiome through the course of untreated and ART-treated SIV infection. Although several genes in the amino acid pathways were significantly decreased following SIV infection, HgdA, KamE, HgCoAd B, and Kce (*p* < 0.05) showed a late recovery signature at the 21 mo ART time-point (Figure 5). This suggests that an incomplete recovery and a sustained loss of butyrate producing genes, particularly in the pyruvate pathway, likely contributed to an increase in inflammation during chronic SIV-ART.

### 3.4. Correlation between Gut Microbiome, Systemic Inflammation, and Leaky Gut Biomarkers during ART-Suppressed Chronic SIV Infection

**Figure 6:**
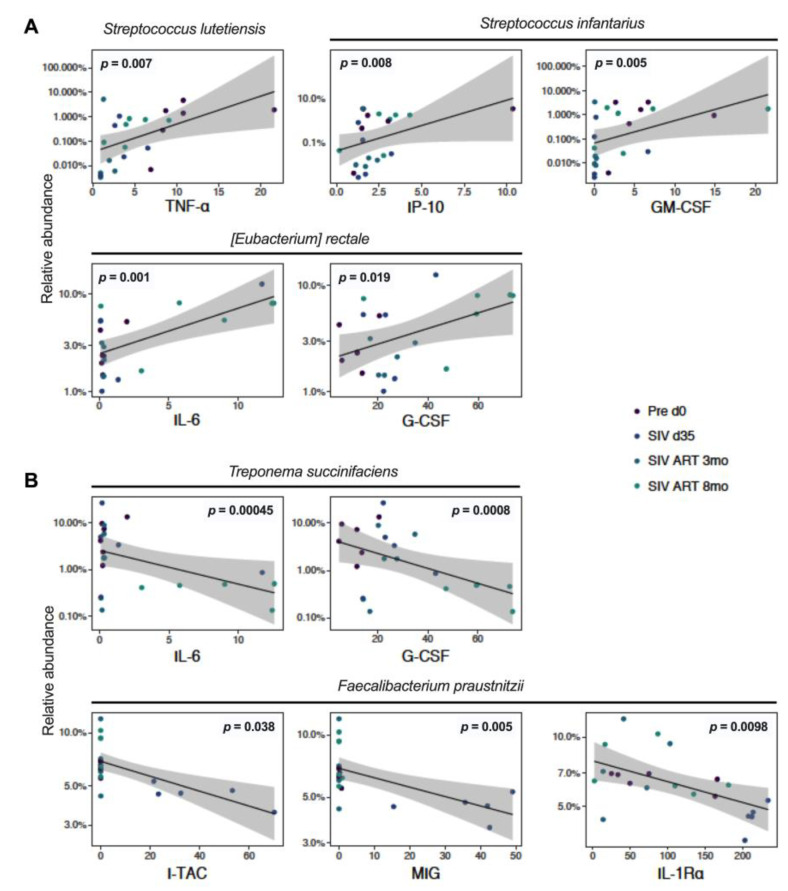
Correlations of gut bacteria with plasma inflammatory cytokines. Logit transformed bacterial relative abundances and plasma cytokine levels with the animal IDs as random effects were correlated using linear mixed effects models to find patterns of host–microbe interactions. (**A**) Species showing a statistically significant positive correlation with inflammatory cytokines; and (**B**) species showing a statistically significant negative correlation with plasma inflammatory cytokines through the course of SIV-ART.

To assess the relationship between the observed changes in gut microbiome, systemic inflammation, and gut epithelial barrier disruption, we analyzed the association between the fecal microbiome composition and plasma levels of cytokines/chemokines and IEBD/MT biomarkers in our cohort of macaques. The levels of *Streptococcus lutiensis*, *S. infantarius*, and *Eubacterium rectale* significantly correlated with plasma levels of inflammatory cytokines including TNF-α (*p* = 0.018), IP-10 (*p* = 0.009), GM-CSF (*p* = 0.0006), IL-6 (*p* = 0.0008), and G-CSF (*p* = 0.0021), thus suggesting a likely role in driving systemic inflammation (Figure 6A).

**Figure 7:**
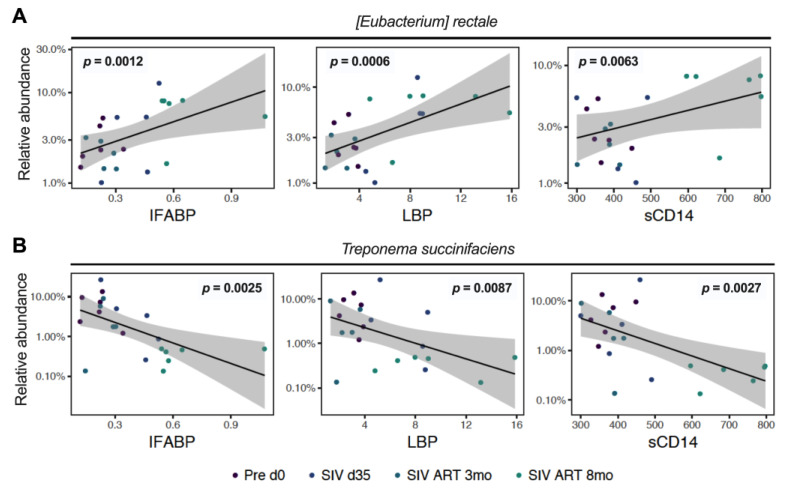
Correlations of gut bacteria with leaky gut biomarkers. Bacterial abundance levels were correlated with plasma levels of an IEBD marker, IFABP, and surrogate markers of MT including LBP and sCD14 using linear mixed effects models to examine the interrelationship between gut microbes and epithelial barrier function. (**A**) Species showing significant positive correlation with IFABP, LBP, and sCD14; and (**B**) species showing significant inverse correlation with IFABP, LBP, and sCD14 through the course of SIV-ART.

A further analysis of correlations with circulating biomarkers of epithelial barrier damage and MT revealed that the relative abundance of *E. rectale* in the fecal microbiome correlated significantly with increases in IFABP (*p* = 0.0011), LBP (*p* = 0.0007), and sCD14 (*p* = 0.0055) during chronic SIV-ART (Figure 7A). Conversely, *T. succinifasciens* levels negatively correlated with IFABP (*p* = 0.0015), LBP (*p* = 0.0097), and sCD14 (*p* = 0.0011; Figure 7B). Together, these data suggest a link between shifts in microbial abundance and leaky gut mediated systemic inflammation.

## 4. Discussion

The discovery of efficient ART regimens has remarkably reduced the rates of HIV-associated mortality and transformed it into a chronic, manageable disease that requires life-long treatment. However, non-AIDS comorbidities including cardiovascular events, neurocognitive disorders, gastrointestinal disease, and neoplasia that are linked to persistent inflammation in long-term treated individuals severely impact the health and lifespan of people living with HIV [46]. Although gut dysbiosis and inflammation persist even with long-term ART in HIV-infected individuals, the role of dysbiosis in the development of chronic inflammation remains unclear. Here, we sought to determine the dynamic relationship between gut microbiome composition and systemic inflammation during acute SIV infection, through the initial immune restorative phase of ART, and during the reemergence of leaky gut and inflammation in chronic SIV-ART. The study findings revealed an early partial recovery of SIV-induced compositional changes and butyrate producing genes in the gut microbiome following ART initiation. However, long-term ART displayed the significant loss of specific bacterial species that correlated with higher levels of plasma inflammatory cytokines, leaky gut, and MT biomarkers, suggesting that despite stable suppression of viremia, chronic SIV infection drives inflammatory changes in gut microbial composition.

In HIV-infected persons, reduced richness of gut microbiota, dysbiosis, and enrichment of pathogenic taxa have been associated with inflammation [17,19,47]; however, gut dysbiosis in SIV-infected macaques is less consistently reported [26,27,31,48]. In our study, despite a high degree of variation between individuals and insignificant differences in overall diversity of the gut microbiome during acute SIV infection, significant changes were observed in bacterial abundance at a species level. This contributed to the observed decrease in the ratio of Firmicutes to Bacteroidetes, in contrast to reports in HIV infection [49,50]. A similar early decrease in F/B ratio, which was progressive and significant during chronic untreated infection, has been reported earlier in the colonic mucosal microbiome [51] and fecal microbiome [29] of SIV-infected rhesus macaques, suggesting that an early decrease in several species of Firmicutes and increase in Bacteroidetes may be specific to nonhuman primates or may have been missed in cross-sectional human studies. The gut microbiota changes following HIV/SIV infection are linked to disruption of the gut epithelial barrier, bacterial translocation of intestinal products from the lumen into the lamina propria, and inflammation [25,43,52,53]. Thus, we intended to systematically address this relationship during chronic SIV-ART to represent the clinical scenario of long-term treated chronic HIV infection. With effective viral suppression during early ART, a trend of greater alpha diversity and lesser variation between animals was reflected by Richness and Shannon indices, which did not reach statistical significance. This coincided with the resolving of plasma inflammatory and leaky gut markers in peripheral blood, indicating the coordinated stabilization of gut microbiome and epithelial barrier function following ART initiation.

At a species level, significant increases in the relative abundance of *A. hadrus* and *L. reuteri* during acute SIV infection in our study was intriguing because, along with *F. prausnitzii*, they are major contributors to butyrate biosynthesis [54,55] and have the potential to improve epithelial barrier function and anti-inflammatory responses. It is likely that in the setting of gut barrier disruption and MT driven by acute SIV infection, this increase further contributed to inflammation. Indeed, supplementing with *A. hadrus* was shown to further aggravate colitis in DSS-treated mice, while no detrimental effect was observed in healthy animals [56], suggesting that the same bacterial species may be beneficial in steady state conditions but may have an inflammatory role in a pathogenic setting. It is also likely that due to low abundance, the increase in *A. hadrus* was inconsequential to the ongoing epithelial barrier disruption and inflammation, as there was a significant decrease in the more abundant anti-inflammatory species including *F. prausnitzii and L. johnsonii* during acute SIV infection. The relative abundance of several members of Ruminococcaceae including *F. prausnitzii* were found to be critically low in HIV-infected persons and inversely correlated with inflammation/immune activation markers [57,58]. Consistent with these observations in HIV infection, in our study the relative abundance of *F. prausnitzii* over the course of SIV-ART was inversely correlated with circulating inflammatory cytokines such as I-TAC and MIG (Figure 6B). Further, the significant decrease in *L. johnsonii* following SIV infection and the sustained loss in four of the six animals throughout the chronic treated infection is consistent with the continued depletion of gut mucosal Th17 cells that we have recently reported in this cohort [40]. The potential role of *L. johnsonii* in the maintenance of Th17 cells and gut epithelial barrier function is further supported by a study in rats showing that administering it as a probiotic lowered gut mucosal inflammation via enhanced Th17 cell bias and elevated IL-23 levels within mesenteric lymph nodes [59].

Overall, the significant changes in the members of Firmicutes during the acute phase of SIV infection in our study were further demonstrated by the reduced abundance of several genes in the butyrate production pathways. Although our study did not measure butyrate levels directly, the specific reduction in several genes involved in the butyrate production pathway suggested a decrease in butyrate in the gut mucosa (Figure 5) and thus reduced availability of this short-chain fatty acid to epithelial and mucosal immune cells. Following 3 months of viral suppression with ART, an early recovery signature in the fecal microbiome comprising of Firmicutes *A. hadrus*, *L reuteri*, and *S. infantarius*, and the Bacteroidetes *Bacteroidales* indicated a trend toward stabilization of the microbiome. Similar findings have been observed in SIV/HIV studies with evidence of mild gut dysbiosis during early acute infection and partial restoration in 2–6 months of ART initiation [29,60].

Interestingly, we found that *T. succinifaciens*, a non-pathogenic carbohydrate metabolizing member of Spirochaetes associated with a high-fiber diet, is progressively reduced in the fecal microbiome despite long-term ART. A similar early decrease of the genus Treponema was noted in SIV-infected macaques by Siddiqui et al. [29] with subsequent recovery by 2 months of viral suppression with ART. The effects of chronic SIV-ART, particularly on *T. succinifaciens* are, however, unknown in HIV/SIV infection. A common constituent of healthy nonhuman primate gut [31,61], *T. succinifaciens* is found in rural or traditional human populations and is lacking or in low abundance in urban populations [62,63,64] likely due to low-fiber diets, or other factors such as frequent antibiotic use. *T. succinifaciens* has been shown to be maintained at a higher abundance in the fecal microbiome of healthy infant rhesus macaques in contrast to those with recurrent diarrhea [65], further indicating their association with healthy gut function. Thus, the linear decrease in our study animals as they progressed through chronic SIV-ART and inverse correlations with inflammatory cytokines IL-6 and G-CSF as well as markers of epithelial barrier damage (Figure 6 and Figure 7) point toward a link between *T. succinifaciens* with gut homeostasis via metabolite production and anti-inflammatory function.

A challenge in most clinical studies is a lack of data on the microbiome composition prior to HIV infection necessitating comparison with healthy control individuals. Given the great variability of bacterial species composition and relative abundance between individuals, it is critical to understand the impact of HIV infection and its treatment on the baseline microbiome composition within individuals beyond the overall differences between patient and control groups. In the SIV-ART model, we had control over many confounding factors such as diet, route of SIV challenge infection, and ART regimen. There were some limitations to our study, such as the small sample size, having all females in our cohort, and use of fecal samples instead of mucosal samples. Despite these limitations, however, our data allowed us to speculate on the potential role of *F. prausnitzii* and *T. succinifaciens* in the maintenance of the epithelial barrier integrity, based on their inverse correlation with circulating markers of enterocyte damage, MT, and inflammatory cytokines (Figure 6B and Figure 7B). In future studies, it would therefore be interesting to investigate whether supplementing these bacterial species can improve gut homeostasis in macaques on long-term ART.

In summary, we have demonstrated that although the initiation of ART enables some recovery of SIV-induced gut microbiome changes, the recovery is partial and not sustained despite the successful long-term control of viremia. This study investigated longitudinal changes in the gut microbiome of macaques through 21 months of ART following SIV infection, which is one of the longest durations of continuous ART evaluated to date in a nonhuman primate model and is equivalent to ~5 years of ART in HIV patients. The progressive changes during the later time-points of ART and their associations with plasma inflammatory cytokines and MT markers are contrary to the earlier speculation that longer ART may be beneficial to restore gut microbiota to healthy levels [29]. These results highlight the need for further research to understand the long-term implications of ART itself on gut microbial communities and intestinal immunity. The identification of bacterial species which inversely correlate with epithelial barrier disruption and MT and are progressively lost during chronic ART could be important for future studies on developing therapeutic interventions to control persistent inflammation in HIV-infected individuals receiving long-term ART.

## Data Availability

Shotgun metagenomics sequence data reported in this paper are deposited in the Sequence Read Archive (SRA) with the accession number PRJNA750063 and the code is available at https://github.com/PennChopMicrobiomeProgram/Rout_Tanes_SIV_ART_microbiome (accessed on 30th July 2021).

## Supplementary Materials

The following are available online at https://www.mdpi.com/article/10.3390/v13081567/s1, Figure S1: Plasma levels of cytokines and chemokines and IEBD/MT biomarkers during SIV infection and long-term ART.
