# Supplementary material for: Gut Microbiome Changes Associated with Epithelial Barrier Damage and Systemic Inflammation during Antiretroviral Therapy of Chronic SIV Infection
Source: Viruses. 2021 Aug 8;13(8):1567. doi: 10.3390/v13081567 (PMC8402875; doi:10.3390/v13081567)
Supplement: Supplementary file 1 [file viruses-13-01567-s001.zip › viruses-1297382-supplementary.pdf]

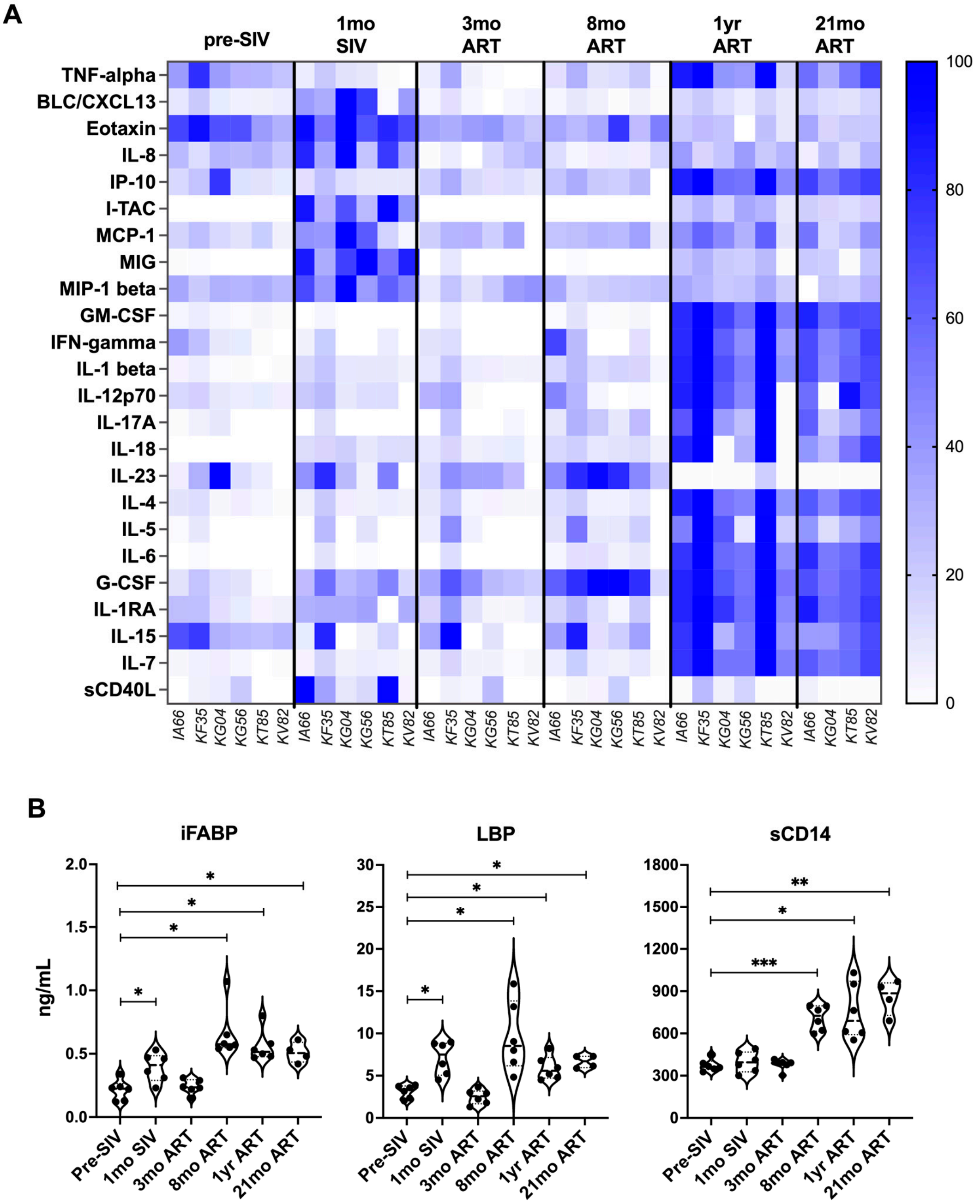

Figure S1. Plasma levels of cytokines and chemokines and IEBD/MT biomarkers during SIV infection and long-term ART. **(A)** Heat map of longitudinal plasma levels of proinflammatory cytokines measured by multiplexed-bead assay in the 6 SIV-infected rhesus macaques. Rows represent each analyte and columns represent each animal at indicated time points. Average cytokine concentration of two technical replicates in pg/ml was normalized to the range for each individual marker so that the fold change represented by each color is consistent across markers. White = lowest value, Blue = highest value for each marker. **(B)** Longitudinal plasma levels of IFABP, LBP, and sCD14 measured by ELISA through the course of ART-suppressed SIV infection. One-way ANOVA with Dunnett's multiple comparisons test was used to determine significant differences from baseline. Asterisks in all panels indicate significant differences between time points (*p < 0.05; **p < 0.01; ***p < 0.001).
